# Anterior ankle impingment syndrome is less frequent, but associated with a longer absence and higher re-injury rate compared to posterior syndrome: a prospective cohort study of 6754 male professional soccer players

**DOI:** 10.1007/s00167-022-07004-4

**Published:** 2022-06-10

**Authors:** Pieter D’Hooghe, Markus Waldén, Martin Hägglund, Håkan Bengtsson, Jan Ekstrand

**Affiliations:** 1 Aspetar Orthopaedic and Sports Medicine Hospital, Sports City Street, Inside Aspire Zone, Al Buwairda St., PO Box 29222, Doha, Qatar; 2 Football Research Group, Linköping, Sweden; 3 Unit of Community Medicine, Department of Health, Medicine and Caring Sciences, Linköping University, Linköping, Sweden; 4 Unit of Physiotherapy, Department of Health, Medicine and Caring Sciences, Linköping University, Linköping, Sweden

**Keywords:** Athletic injuries, Elite, Football, Soccer, Sports, Impingement, Anterior ankle impingement, Posterior ankle impingement, Football medicine

## Abstract

**Purpose:**

To study the epidemiology and return to play characteristics of anterior and posterior ankle impingement syndromes (AAIS and PAIS) over 18 consecutive seasons in male professional soccer players.

**Methods:**

Between the 2001–2002 and 2018–2019 seasons, 120 European soccer teams were followed prospectively for various seasons. Time loss injuries and player exposures were recorded individually in 6754 unique players. Injury incidence and burden were reported as the number of injuries and days absence per 1000 h with 95% confidence intervals (CIs). Injury severity was reported as median absence in days with the interquartile range (IQR).

**Results:**

Out of 25,462 reported injuries, 93 (0.4%) were diagnosed as AAIS (38%) or PAIS (62%) in 77 players. AAIS and PAIS were similar regarding injury characteristics except for a greater proportion of AAIS having a gradual onset (69% vs.47%; *P* = 0.03) and being re-injuries (31% vs. 9%; *P* = 0.01). Impingement syndromes resulted in an overall incidence of 0.03 injuries (95% CI 0.02–0.03) per 1000 h and an injury burden of 0.4 absence days per 1000 h. PAIS incidence was significantly higher than that for AAIS [0.02 (95% CI 0.002–0.03) vs. 0.01 (95% CI 0.005–0.01) injuries per 1000 h (RR = 1.7). The absence was significantly longer in AAIS than in PAIS [10 (22) vs. 6 (11) days; *P* = 0.023]. Impingement syndromes that presented with a gradual onset had longer absences in comparison to impingement with an acute onset [8 (22) vs. 5 (11) days; *P* = 0.014]. Match play was associated with a higher incidence and greater injury burden than training: 0.08 vs. 0.02 injuries per 1000 h (RR 4.7), respectively, and 0.9 vs. 0.3 days absence per 1000 h (RR 2.5).

**Conclusion:**

Ankle injuries are frequent in men’s professional soccer and ankle impingement is increasingly recognized as a common source of pain, limited range of motion, and potential time loss. In our study, ankle impingement was the cause of time loss in less than 0.5% of all injuries. PAIS was more frequently reported than AAIS, but AAIS was associated with more absence days and a higher re-injury rate than PAIS. The findings in this study can assist the physician in best practice management on ankle impingment syndromes in professional football.

**Level of evidence:**

II.

## Introduction

The annual socioeconomic loss of soccer injuries’ costs are over $US 30 billion worldwide [29] with the ankle region as the fifth most commonly injured location, representing 13% of all injuries in soccer [27]. Player contact is a predominant cause of foot and ankle injuries (32–74%), but injuries also occur without player contact [6, 9, 18, 27, 28]. Ankle impingement is recognized as a common source of pain, limited range of motion, and potential time loss in soccer [6, 16, 22].

Ankle impingement is considered a syndrome and can be due to a broad range of pathologies and etiologies. They may present as acute traumatic injuries, but more commonly as gradual-onset conditions due to repetitive stress [11, 22, 28, 30]. While ankle impingement syndromes can be classified in detail according to the anatomical zone into anterior, anterolateral, anteromedial, posterolateral, and posteromedial, most practitioners categorize them, for simplicity, into either anterior ankle impingement syndrome (AAIS) or posterior ankle impingement syndrome (PAIS) [11]. AAIS was first recognized by Morris, who named it “athlete’s ankle” in 1943, and later described as the “footballer’s ankle” by McMurray in 1950 [15, 16, 18]. It is more commonly a bony impingement of tibiotalar osteophytes [21] that has been reported to affect up to 60% of professional soccer players [14]. PAIS, on the other hand, arises more commonly due to forced or repetitive plantarflexion of the ankle [25], which stresses the posterior ankle structures in the tibiocalcaneal interval narrowed by bony structures such as os trigonum, which is present in 7–25% of the general population [3, 8, 20].

The objective of this study was to investigate the epidemiology and return to play characteristics of AAIS and PAIS over 18 consecutive seasons in male professional soccer players.

## Materials and methods

Written informed consents were collected from all participating players in accordance with the Declaration of Helsinki. The general study protocol was reviewed and approved by the UEFA Football Development Division and the UEFA Medical Committee. Individual ethical approval was also obtained from the ethical National review authorities in Denmark, Norway and Sweden (#01-062, #M240-09, and #S-06188).

This is a substudy of a long-term prospective cohort study evaluating men's professional soccer in Europe since 2001, the Union of European Football Associations (UEFA) Elite Club Injury Study (ECIS) [24]. The present study includes data from 18 consecutive seasons of male professional soccer between 2001 and 2019. During the study period, a total of 6754 players from 120 teams representing 25 countries were included. Most of the presented data were collected as part of the ECIS, but since ankle impingement is relatively infrequent, data from five other similar cohorts were also included (the English Premier League, European Artificial Turf Teams, the Swedish First League, the Danish First League, and the Nordic Football Injury Audits) as has been described previously [1].

### Exposure and injury registration

Data collection was undertaken in accordance with the 2006 consensus statement on how to conduct injury surveillance research in soccer [22] and in line with the recent 2020 International Olympic Committee (IOC) consensus statement [2]. The overall study methodology has previously been described in detail [7].

In brief, all first team players in the included teams were invited to participate in the study. At the beginning of every season, teams appointed a contact person within their medical team to be responsible for collecting data and communicating with the study group. Player baseline data were collected at inclusion on an annual basis. All individual player exposures during supervised training sessions and matches were recorded on standard attendance records. Time loss injuries were registered on standard injury cards containing information about the type, location and circumstances of the injury. The appointed contact person reported attendance records and injury cards monthly to the study group that checked the reports and sent feedback to the teams to clarify any missing or unclear data. No specific diagnostic criteria for AAIS and PAIS were used, and it was up to the medical staffs to classify the injuries and refer for imaging or any other consultations needed. All injuries were given a diagnostic code by the study group per the Orchard Sports Injury Classification System (OSICS) 10 [19]. Injury was defined as any physical complaint sustained by a player that resulted from a soccer match or soccer training and led to the player being unable to take a full part in future soccer training or match play. Other relevant injury definitions for this substudy are highlighted in Table 1.

**Table 1:** Injury definitions

Term	Definition
Acute injury	Injury with sudden onset and known cause
Gradual-onset injury	Injury with insidious onset and no known trauma
Minimal injury	Injury causing 0–3 days absence
Mild injury	Injury causing 4–7 days absence
Moderate injury	Injury causing 8–28 days absence
Severe injury	Injury causing > 28 days absence
Re-injury	Injury of the same type and at the same site as an index injury

### Statistical analysis

Data were analyzed using SPSS (IBM SPSS Statistics for Windows, V.26.0, IBM Corp, Armonk, New York, USA). Player demographics are reported with descriptive statistics using mean ± standard deviation (SD) and proportions. Injury incidence was calculated as the number of injuries per 1000 h of exposure ((Σ injuries/Σ exposure hours) × 1000) with corresponding 95% confidence interval (CI). Injury burden was calculated as the number of absence days per 1000 h of exposure ((Σ days absence/Σ exposure hours) × 1000). Rate ratio (RR) was calculated with 95% CI and tested for significance with *Z*-statistics. Injury severity was defined as the number of days of absence and presented as median with interquartile range (IQR). The Mann–Whitney *U* test was used to compare days of absence for different injury categories. The *χ*^2^ test was used to compare the proportions of categorical variables, while Students *t* test was used to compare the mean age, height, and weight between players suffering AAIS and PAIS. The significance level was set at *P* < 0.05.

## Results

During the 18-season period, a total of 3,686,838 h of exposure and 25,462 injuries were reported. 93 AAIS and PAIS syndromes (0.4%) were recorded in 77 players. Eleven players sustained two to four syndrome presentations each during the study period. The right ankle was affected in 50 cases (54%) and the left ankle in 41 cases (44%), while only 2 cases were bilateral (2%). The mean age at the time of the presentation was 25.4 ± 4.2 years. There were no significant differences in baseline characteristics between players who suffered AAIS and PAIS (Table 2).

**Table 2:** Demographics of the study population

	Total injuries (*n* = 93)	AAIS (*n* = 35)	PAIS (*n* = 58)	*P* value
Age, y	25.4 ± 4.2	26.0 ± 4.5	25.1 ± 4.0	n.s
Age group				
< 21 years	12 (13%)	5 (14%)	7 (12%)	n.s
21–25 years	38 (41%)	11 (31%)	27 (47%)	
26–30 years	29 (31%)	11 (31%)	18 (31%)	
> 30 years	14 (15%)	8 (23%)	6 (10%)	
Height (cm)	181.6 ± 6.1	182.2 ± 4.3	181.3 ± 7.1	n.s
Weight (kg)	78.8 ± 6.5	79.4 ± 5.4	78.3 ± 7.2	n.s
Playing position				n.s
Goalkeeper	1 (1%)	0	1 (2%)	
Defender	37 (40%)	17 (49%)	20 (34%)	
Midfielder	34 (37%)	10 (29%)	24 (41%)	
Forward	21 (23%)	8 (23%)	13 (22%)	

*AAIS* anterior ankle impingement syndrome, *PAIS* posterior ankle impingement syndrome

^a^Data are expressed as mean ± SD or *n* (%). (n.s: nonsignificant)

### Injury characteristics

AAIS and PAIS represented 35 (38%) and 58 (62%) of the ankle impingement cases, with the majority having a gradual onset (both *n* = 51, 55%) and occurred during training (Table 3). Sixteen (18%) cases were considered as exacerbation of symptoms. AAIS and PAIS were similar with regard to injury characteristics with the exception of a greater proportion of AAIS being re-injuries [11 (32%) vs. 5 (9%); *P* < 0.01] and having a gradual onset [24 (69%) vs. 27 (47%); *P* = 0.03]. Information about player contact was collected from the 2004/05 season onward and was available for 76 of 93 injuries. Out of those 76 impingement injuries, there was a noncontact injury mechanism in 53 (69%) of the injuries with no difference in the mechanisms between AAIS and PAIS (Table 3).

**Table 3:** Injury characteristics

	Total injuries (*n* = 93)	AAIS (*n* = 35)	PAIS (*n* = 58)	*P* value
Injury side				n.s
Right	50 (54%)	17 (49%)	33 (57%)	
Left	41 (44%)	16 (46%)	25 (43%)	
Bilateral	2 (2%)	2 (6%)	0 (0%)	
Re-injury	(*n* = 93)	(*n* = 35)	(*n* = 58)	< 0.01
No	77 (82.8%)	24 (68.6%)	53 (91.4%)	
Yes	16 (17.2%)	11 (31.4%)	5 (8.6%)	
Mode of onset				0.03
Gradual onset	51 (55%)	24 (69%)	27 (47%)	
Acute onset	42 (45%)	11 (31%)	31 (53%)	
Playing activity				n.s
Training	51 (55%)	23 (66%)	28 (48%)	
Match	42 (45%)	12 (34%)	30 (52%)	
Injury circumstance^b^	(*n* = 76)	(*n* = 26)	(*n* = 50)	n.s
Contact	23 (30%)	7 (27%)	16 (32%)	
Noncontact	53 (70%)	19 (73%)	34 (68%)	

*AAIS* anterior ankle impingement syndrome, *PAIS* posterior ankle impingement syndrome

^a^Data are expressed as *n* (%). (n.s: nonsignificant)

^b^Data collected from the 2004/05 to 2018/19 seasons and available for 76 of 93 injuries

### Return to play and injury severity

**Fig. 1:**
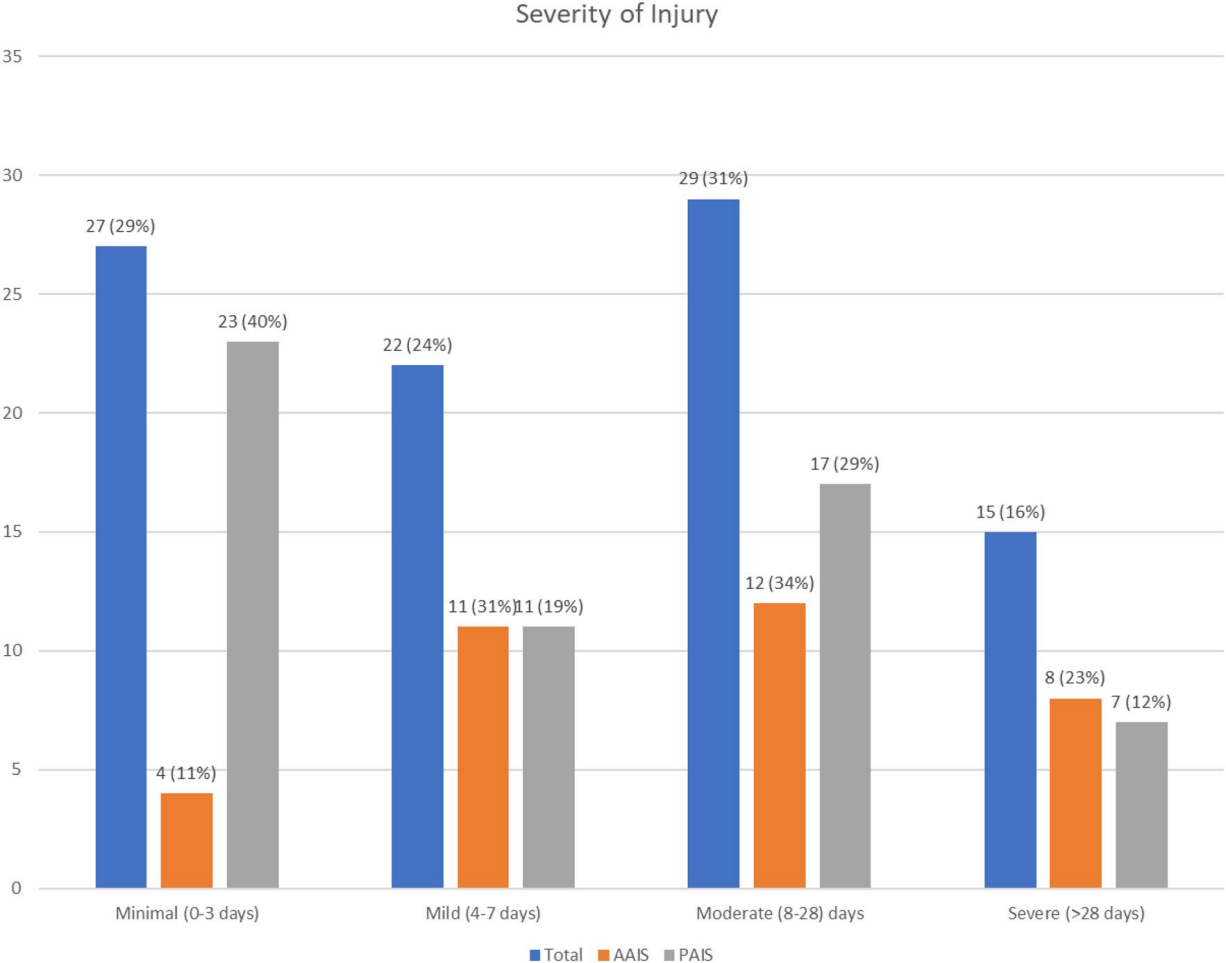
Severity of ankle impingement in male professional soccer players. *AAIS* anterior ankle impingement syndrome, *PAIS* posterior ankle impingement syndrome

The median absence following an ankle impingement injury was 7 (IQR 16) days and 15 (16%) of the injuries were severe with > 28 days on the sidelines (Fig. 1 and Table 4).

**Table 4:** Injury absence and severity

	Total injuries (*n* = 93)	AAIS (*n* = 35)	PAIS (*n* = 58)
Absence days	16.3 ± 23.8	22.7 ± 30.9	12.4 ± 17.5
Range	0–154	1–154	0–90
Median (IQR)	7 (16)	10 (22)	6 (11)
Severity of injury			
Minimal (0–3 days)	27 (29%)	4 (11%)	23 (40%)
Mild (4–7 days)	22 (24%)	11 (31%)	11 (19%)
Moderate (8–28 days)	29 (31%)	12 (34%)	17 (29%)
Severe (> 28 days)	15 (16%)	8 (23%)	7 (12%)

*AAIS* anterior ankle impingement syndrome, *PAIS* posterior ankle impingement syndrome

^a^Data are expressed as mean ± SD or *n* (%)

The median absence was significantly longer in AAIS than PAIS [10 (22) vs. 6 (11) days; *P* = 0.023]. Gradual-onset injuries had significantly longer median absence compared with acute-onset injuries [8 (22) vs. 5 (11) days; *P* = 0.014].

### Injury incidence and injury burden

The overall incidence of ankle impingement was 0.03 injuries (95% CI 0.02–0.03) per 1000 h. The incidence during match play was fivefold higher than during training [0.08 (95% CI 0.06–0.10) injuries per 1000 match hours vs. 0.02 injuries (95% CI 0.01–0.02) per 1000 training hours, RR 4.7 (95% CI 3.1–7.1)].

The incidence of PAIS was almost twofold higher than AAIS [0.02 (95% CI 0.001–0.02) injuries per 1000 h vs. 0.01 (95% CI 0.01–0.01) injuries per 1000 h, RR 1.7 (95% CI 1.1–2.5)]. This difference was mainly seen during match play [0.05 (95% CI 0.04–0.08) vs. 0.02 (95% CI 0.01–0.04) injuries per 1000 match hours, RR 2.5 (95% CI 1.3–4.9)]. No difference was seen during training [0.01 (95% CI 0.01–0.01) vs. 0.01 (95% CI 0.00–0.01) injuries per 1000 training hours, RR 1.2 (95% CI 0.7–2.1)]. A total of 1517 days of absence were reported due to ankle impingement (1046 days due to training injuries and 471 days due to match injuries). This represents an overall injury burden of 0.4 days per 1000 h, with 0.2 days per 1000 h calculated for both AAIS and PAIS (Table 5).

**Table 5:** Injury Incidence and injury burden

	Total injuries (*n* = 93)	PAIS (*n* = 58)	AAIS (*n* = 35)
Injury incidence			
Overall	0.03 (0.02–0.03)	0.02 (0.001–0.02)	0.01 (0.01–0.01)
Training	0.02 (0.01–0.02)	0.01 (0.01–0.01)	0.01 (0.00–0.01)
Match	0.08 (0.06–0.10)	0.05 (0.04–0.08)	0.02 (0.01–0.04)
Injury burden			
Overall	0.4	0.2	0.2
Training	0.3	0.1	0.2
Match	0.9	0.5	0.4

*AAIS* anterior ankle impingement syndrome, *PAIS* posterior ankle impingement syndrome

^a^Data are expressed as number of injuries and absence days per 1000 h with 95% confidence interval

## Discussion

The principal findings of this study were that PAIS occurred more frequently than AAIS and presented relatively more frequently with a gradual onset, whereas AAIS led to longer average absence and more re-injuries.

We calculated from Tables 4 and 5 that the total nr of absence days for AAIS was 770 and for PAIS 696. The number of days the players are absent is the key message that the coaches are listening to and the fact that PAIS has almost the same consequences for a team as the more well-known AAIS, might be a new and useful information.

### Return to play

The present study found that soccer players with ankle impingement were able to return to play (RTP) at a median of 7 days (10 days in AAIS and 6 days in PAIS, respectively) following the injury occurrence, with only 16% of the injuries requiring longer than 28 days to recover. Importantly, our data included both injuries with nonsurgical and surgical treatment, whereas a few other studies have reported RTP details on surgically treated PAIS in soccer players exclusively. For example, Lopez-Valerio et al. [12] reported on 20 professional soccer players in Brazil who were treated arthroscopically for PAIS, and reported a mean RTP to the previous level of activity at an average of 46.9 days. Similarly, Calder et al. [4] reported an average of 34 days to return to training and 41 days to RTP in elite soccer players following arthroscopic surgery of PAIS. They also reported a sooner return in players with soft tissue rather than bony impingements. Finally, Kudaş et al. [10] reported an average RTP at 36 days of nonsurgical treatment in elite Turkish soccer players with PAIS. Murawski and Kennedy [17] reported RTP at a mean of 7 weeks (5–13 weeks) post-arthroscopic debridement of AAIS in a mixed population. The days of absence in the current study are fewer than reported in the literature and are to be interpreted with caution due to the lack of treatment data and detailed pathology (e.g., soft tissue vs bony impingement).

In the present study, AAIS led to significantly greater days of absence than PAIS. While such a comparison was not the main objective of this study, it could potentially be explained by the treatment strategy. In a previous study, AAIS had a higher probability of failure of nonsurgical management and thus requiring surgery, in comparison to 60% chance of success in PAIS after nonsurgical treatment [20]. Another possible cause for our findings is that soft tissue impingement, which is more common in PAIS, results in quicker recovery than bony causes of impingement. In the aforementioned series of 27 professional soccer players who underwent PAIS arthroscopic treatment, Calder et al. [4] reported a quicker return to training in soft tissue impingement in comparison to bony impingement (28 days vs. 40 days, respectively). Moreover, arthroscopic management of PAIS is considered to be safer in terms of nerve injuries than anterior arthroscopy needed for AAIS [12, 20, 23, 26].

### Injury incidence

The overall incidence of symptomatic ankle impingement was 0.03 cases per 1000 h, with PAIS being 1.7 times more common than AAIS. Interestingly, the incidence was almost five times higher during match play compared with training. This could be attributed to the more unpredictable and aggressive style of play during matches, and similar findings were described by Lubberts et al. [13] who found that syndesmotic ankle injuries in professional soccer players were 13 times more frequent during match play compared with training.

Re-injury was identified as a repeated period of time loss due to AAIS or PAIS within the same season as a previous identical injury. In this paper, the re-injury variable was based on what was reported by teams. Teams were instructed to report injuries as re-injuries when a player suffered a second period of absence due to an injury of the same type and affecting the same location as a previous index injury.

### Etiology of impingement syndromes

Multiple theories have been proposed regarding the etiology of AAIS, and a significant contribution to our understanding of the pathology has been done by Tol and van Dijk [24] in the 1990s and early 2000s. The earlier theory, first described by McMurray in 1950, attributed it to the traction forces on the anterior ankle capsule during forced plantar flexion, leading to the formation of anterior tibiotalar osteophytes and subsequent soft tissue proliferation and impingement [15, 25]. However, this theory has its limitations and has been disputed in favor of repetitive dorsiflexion and microtrauma [10, 15, 29]. An anatomical study by Tol and van Dijk [25] found the attachment of the anterior capsule to be on average 4 mm proximal to the cartilage. Likewise, a few other studies found the capsule to also be around 6 mm proximal to the site of the tibial spur, and arthroscopic examination has shown the spurs to be within the ankle joint and not in the joint capsule. Our results that the majority of ankle impingements had a gradual onset and AAIS was more common than PAIS support the subsequent theory of direct repetitive microtrauma and the findings of the biomechanical study by Tol et al. [25], where they concluded that the impact of a soccer ball to the anteromedial side of the cartilaginous rim generated sufficient forces to cause damage [5, 30].

### Prevention and early recognition of impingement syndromes

Walls et al. highlighted the importance of injury and re-injury prevention and early recognition of ankle injuries in soccer players to optimize outcomes and reduce absence from sport [29]. Proper warmup, stretching, sufficient recovery, proprioception, and neuromuscular exercises are paramount for injury prevention. Additionally optimizing field conditions can further reduce injury incidence, especially non-contact injuries. Artificial turf, longer cleats, and dry hardened turf can increase the shoe–surface friction and thus increase the risk of ankle injuries [29]. An 18% recurrence rate and total a of 1517 days of absence due to ankle impingement were shown by our results, which represent an injury burden of 0.4 days absence per 1000 h. Hence, more organized efforts to prevent ankle impingement syndromes are required.

The clinical relevance of the findings in this study is that the provided epidemiological data on AAIS and PAIS in elite soccer can guide the clinician toward the best evidence-based ankle impingment management.

Unfortunately, this study presents several limitations. First, the injury form did not record the diagnostic tests and examination findings of all players and was not able to record data of all potentially important variables. The diagnosis was made by the medical teams of each soccer team and is consequently subject to biases, different experiences of different physicians, and availability of resources. Second, there was no information on how players with either AAIS or PAIS were treated, and if they had associated arthroscopic findings if they underwent surgical treatment. The type of impingement, whether bony or soft tissue, was also not recorded. Third, we did not capture data on the players’ medical history, such as ankle instability or fractures. Fourth, AAIS led to time loss in our study compared to PAIS. This might also be because AAIS more frequently requires surgery. Fifth, we acknowledge the limitation of our injury definition that could clarify why PAIS was a more frequent cause of time loss in professional football, while we are unaware of the total prevalence of symptomatic ankle impingement in the cohort, since many symptomatic AAIS and PAIS may not be captured when using a time loss injury definition given that players may have symptoms but are not taken out of play. Sixth, even though the study sample of professional players was large, some sub-analyses were limited by a small number of injuries and there is a risk of type 2 error. Finally, we utilized in-season time loss as an indicator of the severity of the injury. Consequently, injuries with off-season rest, treatment, or surgery might be missed.

## Conclusion

The overall incidence of symptomatic ankle impingement in the current study was 0.03 injuries per 1000 h and resulted in an injury burden of 0.4 days absence per 1000 h. PAIS was 1.7 times more frequent than AAIS, but days of absence was significantly greater for AAIS in comparison to PAIS and AAIS had a higher re-injury rate than PAIS.
